# Increase of Albinistic Hosts Caused by Gut Parasites Promotes Self-Transmission

**DOI:** 10.3389/fmicb.2018.01525

**Published:** 2018-07-10

**Authors:** Shuqian Tan, Yang Wang, Pingping Liu, Yang Ge, Aomei Li, Yongjie Xing, David M. Hunter, Wangpeng Shi

**Affiliations:** ^1^Department of Entomology and MOA Key Lab of Pest Monitoring and Green Management, College of Plant Protection, China Agricultural University, Beijing, China; ^2^Independent Researcher, Canberra, ACT, Australia

**Keywords:** *Locusta migratoria*, intestinal parasite, albinism, positive transmission strategy, dopamine pathway

## Abstract

*Paranosema locustae* is a gut parasite that has been applied widely in the control of grasshoppers in many parts of the world. Usually, *P. locustae* is transmitted horizontally via passive modes under natural conditions but in the current study, a positive transmission strategy of *P. locustae* was demonstrated. First, infection by *P. locustae* resulted in the cuticula of infected *Locusta migratoria* nymphs to become lighter in color: normally only a small proportion of locusts are pale with most either being partly or mostly black; but locusts infected with *P. locustae* became pale. And it was found that the change to pale occurred even among uninfected black and partly black nymphs reared with infected locusts. The eumelanin of the thorax and abdomen of infected individuals decreased significantly, as did the level of dopamine. In addition, there was a decrease in phenol oxidase activity and the expression of *henna* and *pale*, which are involved in the synthesis of cuticle melanin, decreased. What is the ecological significance of this increase in light-colored hosts caused by *P. locustae*? We discovered that light-colored locusts were more susceptible to the microsporidian pathogen than dark-colored individuals were, because of their weaker melanization. Phenol oxidase activity in pale locusts was lower than that of black locusts, but the *serpin* expression level of pale locusts was higher than that of black individuals. When examined for infection, it was found that initially uninfected nymphs had picked up *P. locustae* infections indicating that infections are readily passed from one pale locust to another. The infection rate of healthy locusts reared with light-colored locusts infected with *P. locustae* was 100% which was more than with black-colored ones. The increase in albinistic locusts clearly promoted the prevalence of *P. locustae* in the total population. In conclusion, these results elucidated a new strategy of positive self-transmission in *P. locustae*.

**Importance**:

Mother Nature always grants wisdom to her creatures and feeds them carefully. This wisdom is particularly apparent in the relationships between two interacting species. In this study, our team focused on the interaction between *L. migratoria* and *P. locustae*. In a previous study, it was found that *L. migratoria* isolate infected individuals, reducing avoiding the spread of *P. locustae*, in a previous study. The solitary, pale individuals infected by *P. locustae* were left behind as locust groups marched ahead, leading to a kind of behavioral immunity in the insects. Here, we reported that *P. locustae* promotes pigmentation loss in *L. migratoria*, causing a larger proportion of light-colored individuals, and these lighter individuals which possessed weaker immunity against pathogens. This strategy is advantageous to *P. locustae*, as it promotes its propagation and spread. These extraordinary abilities of *L. migratoria* and *P. locustae* have accumulated over millennia of years of interaction.

## Introduction

Numerous entomopathogens (pathogenic fungi, pathogenic bacteria and insectivorous nematodes) transmitted in host populations follow the classical vertical and horizontal transmission modes (Bright and Bulgheresi, 2010; Watanabe et al., 2014; Fisher et al., 2017). However, most horizontal transmission routes are passive. In classical transmission modes, pathogens or spores are generally entrained by wind, water flow and host or even non-host organisms (Liu et al., 2016). This requirement means that environmental factors (humidity, environment temperature, and ultraviolet intensity, etc.), which are intermediaries in the transmission process, can influence the transmission efficiency of pathogens (Thomas et al., 1996; Kassa et al., 2004). In fact, multiple studies have demonstrated that *P. locustae* circulates over greater distances and for longer periods in certain environmental conditions (Shi et al., 2009; Miao et al., 2012). Although both horizontal and vertical transmission mechanisms exist in *P. locustae* (Henry and Oma, 1981; Raina et al., 1995; Lange, 1997), the evidence to date has not demonstrated a positive transmission strategy in *P. locustae*, but we provide evidence for such transmission here.

Color polymorphisms are found in many insects, and phenotypic plasticity has been shown to be primarily controlled by interactions between environmental and genetic factors (Forsman and Appelqvist, 1999; Mallet and Joron, 1999). Variations in the color of parts of the body provide crucial information for studies of insect evolution and classification. Grasshoppers in the genus *Chitaura* (Orthoptera: Acrididae) have at least 20 parapatrically distributed color forms in Sulawesi and the Moluccas (Butlin et al., 1998). These color morphs differ in several traits, including behavior, thermal biology and body size (Ahnesjö and Forsman, 2003). Moreover, immunoreaction was confirmed and may be genetically or developmentally linked with color pattern. In *Tetrix undulata*, darker patterning may be a direct consequence of selection for resistance to the parasite *Leiophora innoxia*, due to a higher encapsulation response in darker-patterned individuals (Civantos et al., 2005). The melanic morph had more resistance to the parasite than the non-morph because of its thicker cuticle, higher expression of immunity genes, higher numbers of circulating hemocytes and upregulated cuticle phenol oxidase (PO) activity concomitant with pathogens invasion (Dubovskiy et al., 2013).

Several kinds of phenotypes with various color forms are well known to exist in gregarious locusts, with gregarious locusts usually being darker in color than solitaries. Coincidentally, crowded locusts are considered to possess stronger immunity than solitary locusts (Wang et al., 2013). However, whether *P. locustae* can cause color pattern changes in *L. migratoria* and the nature of the relationship between color forms and pathogen transmission has remained unknown until now. Thus, this study examined the variation of cuticle color in the locust *L. migratoria* after infection with *P. locustae*, and the effects of exposure to infected locusts on the cuticle color of healthy black locusts. We also examined the differences in immunity factors related to pathogen resistance, such as PO activity, gene expression level of *serpin*, level of propagation and spread of the pathogen, between light- and dark-colored locusts.

## Materials and Methods

### Insects and Infection (Tan et al., 2015)

The locusts (*L. migratoria*) used for this study were from a laboratory colony originating from a stock obtained for the Key Biocontrol Laboratory for Locusts in Beijing, China. Groups of 80–100 hatchlings were reared in a metallic cage (50 cm high by 15 cm diameter) kept in a climate controlled cabinet (PXZ-430B, Ningbo Jiangnan Instrument Factory, China) under a long-day photoperiod (16 h light/8 h darkness cycle) at 30 ± 2°C and 75% relative humidity. Locusts were fed wheat seedlings.

The original stock of *P. locustae* was obtained from the U.S. Department of Agriculture, Agricultural Research and Service, Rangeland Insect Laboratory, Montana State University, Bozeman. The *P. locustae* used in this experiment was propagated by the experimental factory at China Agricultural University, Beijing. According to the inoculation method described previously (Tan et al., 2015), the 1-day old third-instar nymphs were starved for 24 h (the nymphs were still in third-instar) and inoculated with 2 × 10^3^, 2 × 10^4^, or 2 × 10^5^ *P. locustae* spores. Cages of inoculated and of untreated nymphs (controls) were then kept in conditions described above: while the locusts were initially in the third instar, at the rearing temperature of 30 ± 2°C, they reached the fourth instar within 1 week and the fifth instar within 2 weeks so that the locusts were in the fifth instar by the time the experiment was completed.

### Patch Analysis

Pictures of the pronotum were taken by a CCD (Charge Coupled Device) imaging system (exposure: 195.8; gain: 1.0×; saturation: 2; gamma: 1.00) and ImageJ software was used to determine their grayscale level and the area of their melanin patches which gave the proportion of the pronotum or abdomen with spots. Several individuals were tested for each treatment in three separate tests. We also measured the height of the patches on the abdomens of several fifth-instar locusts in each treatment in three separate tests.

### Preparation of Locust Integument, Fat Body and Hemolymph

Hemolymph was collected in 1 mL of ice-cold saline and then centrifuged at 1000 ×*g* at 4°C for 3 min to separate the hemocytes, which were washed three times with 1 mL of locust saline (Wang et al., 2013). The hemolymph samples collected for enzyme activity analysis were whole blood and were tested as soon as collected. The insect tissues (fat body and integument) were dissected immediately after the hemolymph collection. All samples were directly frozen in liquid nitrogen until RNA sample preparation.

### Melanin and Dopamine Content Analysis

For one sample, 5 eviscerated nymphs were pooled and homogenized in distilled water. A blank tube contained 100 μL sample and 900 μL Soluene-350 (PerkinElmer, United States). After ultrasonication for 5 min, the tubes were vortexed and placed in a boiling water bath for 30 min. The cooled tubes were revortexed and heated for an additional 15 min in boiling water. The cooled tubes were centrifuged at 10,000 rpm for 10 min. Samples were analyzed for absorbance at 650 nm, which provided a measurement of eumelanin (Häkkinen et al., 2001).

The blank tube contained 100 μL somatic tissue suspension and 400 μL 8 M urea/1 M NaOH solution. All alkali-soluble melanin was completely dissolved by shaking for 10 min. The tubes were centrifuged at 10000 rpm for 10 min. We then mixed the supernatant with 500 μL chloroform and then vortexed and centrifuged the mixture at 4000 rpm for 10 min. We then transferred the supernatant into a new tube, which was then centrifuged at 10000 rpm for 10 min, and 150 μL of the resulting liquid was analyzed for absorbance at 400 nm (Ozeki et al., 1996).

The dopamine concentration of each sample was measured in the hemolymph and integument using an insect dopamine (DA) ELISA Kit (TSZ, United States). The values were represented as the means (±SE), and statistical significance was determined by using an independent-samples *T*-test with SPSS 16.0 software.

### Phenol Oxidase Activity Analysis

The PO activity in the reaction mixture was measured as described previously (Jiang et al., 2003), and used dopamine as a substrate (Jiang et al., 2003). PO activity was measured by adding 200 μL of 2 mM dopamine in 50 mM sodium phosphate, pH 6.5, to each sample well. Absorbance at 470 nm was then monitored continuously using a microplate reader (BioTek, United States). One unit of PO activity is defined as an increase in 0.001 absorbance units/minute.

### Survival Analysis

The third-instar larvae with the same ecdysis time were classified using the amount of pigmentation on the surface of the cuticle into two morph types: black (black and intermediate) and pale. Then, the locusts (standard laboratory population consisting of either one or both phenotypes) were inoculated with *P. locustae* (200,000 spores/locust). The experiment was performed in triplicate. The survival curves were compared using Kaplan–Meier and Cox’s proportional hazards model, which has been used to assess variables that affect locust survival. The *P*-value threshold was adjusted by Bonferroni correction. SPSS 16.0 software was used in all statistical analyses.

### Feces Collection From Locusts and Separation of *Paranosema* Spores

Every 2 days, feces were collected from the locusts infected with *P. locustae* (2 × 10^5^ spores/locust), and spores were separated from the feces using the method described by Shi and Njagi (2004). Then, 0.5 g of feces was collected, homogenized with a mortar and pestle, and suspended in distilled water, and the resulting suspension was filtered through folded medical gauze. The filtrate was centrifuged at 1,000 rpm for 3 min, and the supernatant liquid was centrifuged again at 6,000 rpm for 10 min. Spores of *P. locustae* were counted on a hemocytometer (YA-XB100, Hengtai, Yancheng, China) under a compound microscope (B102, OPTEC, Chongqing, China).

### Gene qRT-PCR Assay

Three biological replicates (3–5 individuals per replicate) were pooled for three parallel technical analyses. The mRNA of each sample was extracted from the dissected tissues (fat body and integument) and collected hemocytes using Invitrogen TRIzol Reagent (Life Technologies, United States). The first-strand cDNA was synthesized using a FastQuant RT Kit (with gDNase) (Tiangen Biotech, China). The cDNA was serially diluted 5-fold, and the dilutions were used for analyzing the PCR efficiency of the primers. The preparation of the RT-PCR mixture was conducted as instructed by the manual of the SYBR^®^ Premix Ex TaqTM (Tli RNase H Plus) (Takara, China). The reactions were performed on a 7500RT (ABI, United States) using the two-step method and completed with a melting curve analysis program. The specificity of the qRT-PCR primers was confirmed by the melting curve and by sequencing of the qRT-PCR products. The β*-actin* sequence of *L. migratoria* (GenBank accession no: AF370793) was used as the internal control. All primers are presented in Supplementary Table S3. Values were represented as the means (±SE), and statistical significance was determined by using an independent-samples *T*-test with SPSS 16.0 software.

## Results

### Variation in Locust Cuticle Color After Infection

**FIGURE 1:**
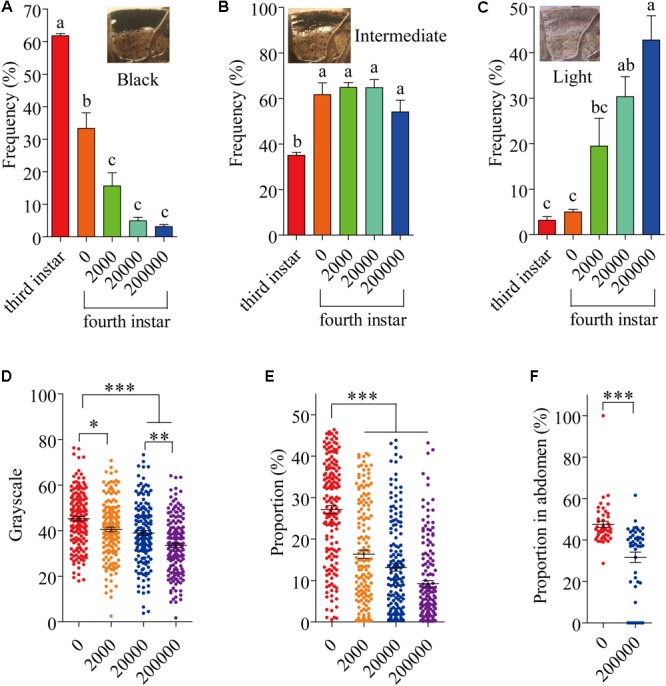
The variation of locust cuticle color. **(A)** Pronotum of black locusts, the proportion of black locusts in experimental populations on day 8 (fourth instar). **(B)** Pronotum of intermediate locusts, the proportion of intermediate locusts in experimental populations on day 8 (fourth instar). **(C)** Pronotum of light locusts, the proportion of light locusts in experimental populations on day 8 (fourth instar). **(D)** The gray level of the pronotum on day 8 (fourth instar) (*N*_0_= 165; *N*_2000_= 1550; *N*_20000_= 160; *N*_200000_= 161). **(E)** The proportion of the pronotum with spots on day 8 (fourth instar) (*N*_0_= 165; *N*_2000_= 155; *N*_20000_= 160; *N*_200000_= 161). **(F)** The proportion of the abdomen covered in spots on day 16 (fifth instar) (*N*_0_= 45; *N*_200000_= 45). The digits below the *x*-axis represent the dose (spores/locust) of *P. locustae* injected into the locust. Values (mean ± SE) with different letters are significantly different (*P* ≤ 0.05) Tukey’s Multiple Comparison Test. Values (mean ± SE) with the “^∗^”, “^∗∗^”, and “^∗∗∗^” are significantly different in 0.05, 0.01, and 0.001 level, respectively (Kruskal–Wallis test). The “third instar” and “fourth instar” were healthy laboratory locusts as a check.

Several different phenotypes exist in laboratory populations of locusts. These phenotypes are usually differentiated into three types (black, intermediate and light) (**Figures 1A–C**). Usually, 61.82% of 3 instar individuals were black phenotype in laboratory populations (**Figure 1A**); 33.33% of 4 instar individuals were black phenotype (**Figure 1A**). By 8 days after infection with *P. locustae*, by which time the locusts had reached the fourth instar, the number of dark-colored locusts was significantly reduced (*P* < 0.05) as spore concentrations increased (**Figure 1A**), while the number of light-colored locusts increased (**Figure 1C**). However, the number of intermediate grasshoppers remained relatively constant (**Figure 1B**). Compared to the third-instar locusts, the fourth-instar individuals were lighter (dark-colored locusts were fewer and light-colored were more numerous), overall (**Figures 1A–C**). This phenomenon occurred on the pronotum and expanded to the whole body with increasing infection time (Supplementary Figure S1). In addition, the results showed that the grayscale value of the pronotum declined with increasing spore concentration (**Figure 1D**); the size of the dark, spotted area on the top half of the pronotum decreased (**Figure 1E**). However, the variation of the spots on the pronotum of fifth-instar nymphs (16th day) was inconspicuous (Supplementary Figure S2).

The fifth-instar locusts were divided into two types by pronotum phenotypic analysis in our experiment: black and light (Supplementary Figure S1). These types allowed a convenient description of the results obtained in this study. At 16 days after the experiment began, by which time the locusts had reached the fifth instar, there were significantly fewer (*P* < 0.05) infected locusts were black-colored compared to uninfected controls, with infected locusts being mainly light-colored (Supplementary Figure S1B). However, infected locusts had a significantly (*P* < 0.05) lower proportion of the abdomen covered in dark spots compared with infected individuals (**Figure 1F**).

### Reduction of Cuticular Melanin in Locusts Infected With *P. locustae*

Because melanin is responsible for cuticle pigmentation, we assayed the level of melanin (eumelanin, total melanin and alkali solubility of melanin) in the thoracic and abdominal cuticle of the migratory locust. The amounts of thoracic eumelanin at 8 and 16 days after locusts were inoculated with 200,000 spores were reduced significantly, by 33.0% (*P* < 0.05) and 75.9% (*P* < 0.05), respectively (**Table 1**). A decrease in thoracic eumelanin also occurred in locusts infected with a lower concentration of *P. locustae* (20,000 spores/locust) (*P* < 0.05). A similar decline also occurred in the abdominal cuticle of locusts infected by *P. locustae* (**Table 1**).

**Table 1:** Impact of *P. locustae* infection on eumelanin (EM) in nymphs.

Instar	Inoculation dose	Eumelanin (EM), OD/g	
		Thorax	Abdomen
Fourth instar (8 days post-inoculation)	Untreated	5.00 ± 0.38a	3.27 ± 0.27b
	20000 spores/locust	3.10 ± 0.19b	5.20 ± 0.40a
	200000 spores/locust	3.35 ± 0.36b	1.93 ± 0.27c
Fifth instar (16 days post-inoculation)	Untreated	19.67 ± 4.00a	10.25 ± 1.04a
	20000 spores/locust	7.25 ± 1.36b	7.17 ± 1.66ab
	200000 spores/locust	4.75 ± 1.55b	3.89 ± 0.59b

*Data are mean ± SE levels of eumelanin (EM) in integument of fourth- and fifth-instar nymphs after inoculation with *P. locustae*. Any two treatments with the same lower-case letter are not significantly different at α = 0.05 (one-way ANOVA). The time of third instar: about 7 days; the fourth instar: about 8 days; the fifth instar: about 10 days in the laboratorial conditions.*

Total melanin (TM) and alkali-soluble melanin (ASM), which includes some non-eumelanin compounds, are also general indexes for melanin, and both these indexes were reduced after infection (Supplementary Tables S1, S2). The value of TM was significantly reduced in the abdomen at the 8th day after infection by 200,000 spores (*P* < 0.05) (Supplementary Table S1). By the time of *P. locustae* infection, the third-instar nymphs had developed into adults, and the levels of ASM were substantially reduced (Supplementary Table S2).

### Reduction in Dopamine in the Cuticular Tissue of Infected *Locusta migratoria*

**FIGURE 2:**
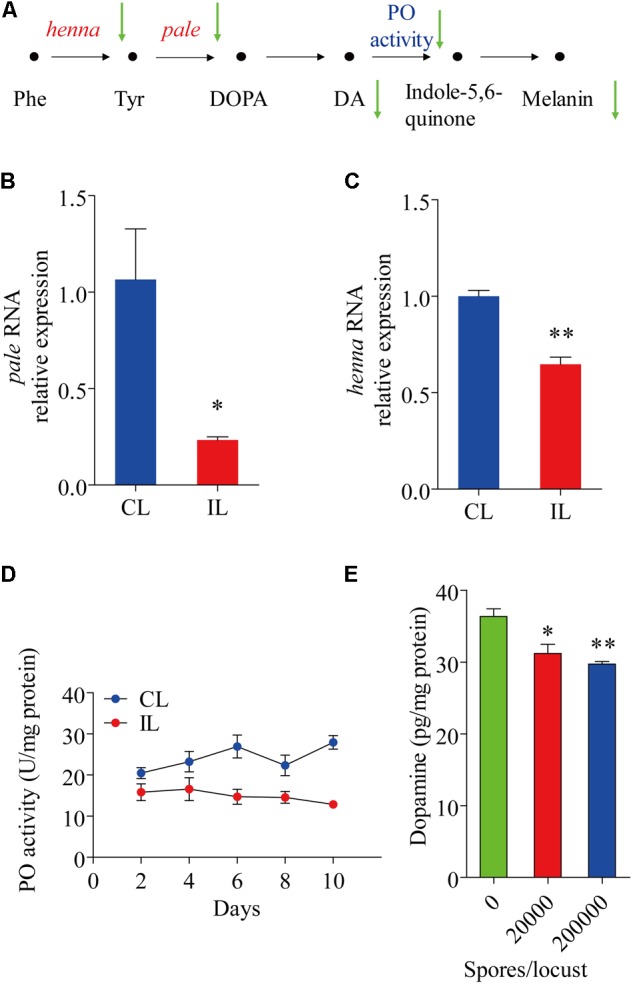
The effect of *P. locustae* on the dopamine pathway in *L. migratoria*. **(A)** The pathway of melanin formation in *Drosophila melanogaster*. Phe, Phenylalanine; Tyr, L-tyrosine; DOPA, 3,4-dihydroxy-L-phenylalanine; DA, dopamine; PO, phenol oxidase. The green arrows showed decrease (IL vs. CL). *henna* and *pale* **(B)** analysis of *pale* RNA relative expression. **(C)** Analysis of *henna* RNA relative expression. **(D)** Spatiotemporal dynamics of phenol oxidase activity affected by *P. locustae.* **(E)** Dopamine content in integument. CL, Control Locust; IL, Infected Locust. Values (mean ± SE) with the “^∗^” are significantly different (*P* ≤ 0.05) from uninfected controls (infected 0 spores/locust) (Independent-Samples *T*-test) and with the “^∗∗^” are significantly different (*P* ≤ 0.01).

The expression of *henna* and *pale*, which encode phenylalanine hydroxylase and tyrosine hydroxylase, respectively, were analyzed. The expression of *pale* was reduced by 0.766-fold (*t* = 3.167, df = 4, *P* = 0.034) (**Figure 2B**), while that of *henna* was reduced by 0.353-fold (*t* = 7.452, df = 4, *P* = 0.002) (**Figure 2C**). Compared with uninfected healthy locusts, infected locusts had significantly lower Phenyl Oxidase enzyme activity starting from day 2 after infection by *P. locustae* (200,000 spores/locust) (**Figure 2D**). The dopamine in the cuticle was also significantly decreased (*t* = 3.095, df = 4, *P* = 0.036) at the lower infection level (20,000 spores/locust) (**Figure 2E**). On the 8th day after infection with 200,000 spores/locust, the dopamine content in the locust cuticle was tested and found to be significantly decreased (*t* = 5.922, df = 4, *P* = 0.004) (**Figure 2E**). In *Drosophila melanogaster*, cuticle melanization is closely related to catecholamine metabolism, and the key precursor of insect melanin is dopamine (Marika et al., 1996) (**Figure 2A**). The decreases in *pale* and *henna* expression and Phenyl Oxidase activity in locusts infected with *P. locustae* reduced DA and melanin production (**Figure 2A**).

### Complementary Exogenous Dopamine Recovered Cuticle Pigmentation

**FIGURE 3:**
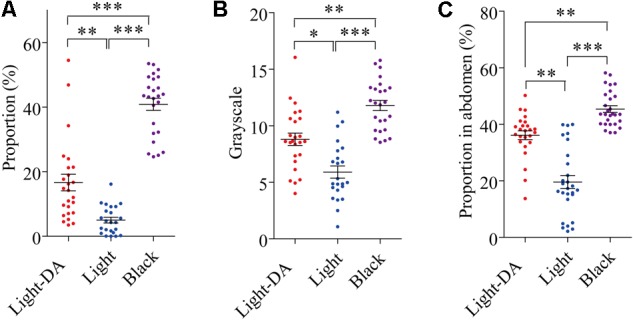
The variation of locust cuticle color after supplementing exogenetic dopamine. Light colored locusts (Light-DA) were injected with 1 μg/μL dopamine hydrochloride solution and these were compared with Light and Black colored locusts not given dopamine: there were 23–28 locusts in each group. **(A)** The gray level of the pronota on the 8th day. **(B)** The proportion of the pronotum covered in spots on the 8th day. **(C)** The proportion of the abdomen covered in spots on the 16th day Light-DA: injection with 1 μg/μL dopamine hydrochloride solution. Values (mean ± SE) with the “^∗^”, “^∗∗^”, and “^∗∗∗^” are significantly different in 0.05, 0.01, and 0.001 level, respectively (Kruskal–Wallis test).

After injection with 1 μg/μL dopamine hydrochloride solution, the grayscale level and the area of the pronotum covered in dark spots on the 8th day (fourth instar) and the area of abdomen covered in dark spots on the 16th day (fifth instar) were analyzed. Those treated with dopamine darkened to be intermediate between the light and black in all three measures (**Figures 3A–C**).

### Light-Colored Gregarious Locusts Showed a Lower Tolerance to *P. locustae* Infection Than Black-Colored Ones Did

**FIGURE 4:**
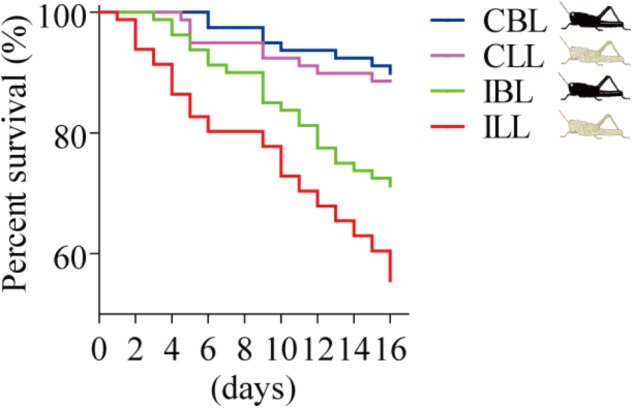
Percent survival after *P. locustae* infection of various phenotypes of the laboratory population. CBL, control locusts of black phenotype (*N* = 79); CLL, control locusts of light phenotype (*N* = 79); IBL, infected locusts of black phenotype (*N* = 80); ILL, infected locusts of light phenotype (*N* = 81). Kaplan–Meier method in SPSS 13.0 was used to analyze locust survival data. The experiment was performed in triplicate, and the data of all repeats were similar.

We compared the survival of uninfected and infected locusts of the two phenotypes (black and light). The results showed the same trend, with the survival of infected locusts that were light being lower than that of infected locusts that were black (χ*^2^*= 4.432, *p* = 0.035, log-rank test) in that survival of light colored locusts declining to 56% by the 16th day post-infection (**Figure 4**).

### Immunity Factors Related to Melanization Were Stronger in the Black Phenotype Than in the Light Phenotype

**FIGURE 5:**
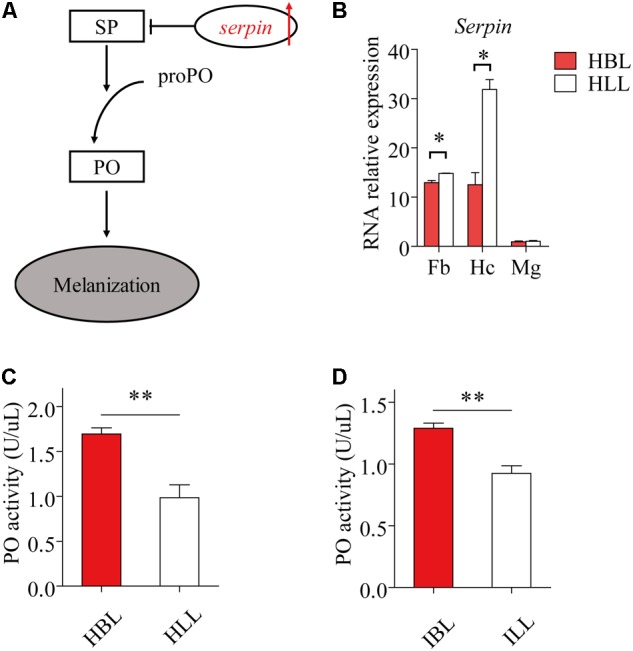
The immunity factors of melanization in light and black locusts. **(A)** The schematic diagram of melanization. The red arrow shows increase (IL, infected locusts vs. HL, healthy locusts). SP, serine protease; PO, phenol oxidase; proPO, prophenoloxidase. **(B)** *Serpin* expressed transcripts between normal black and light locusts in main immune tissues (Fb, fat bodies; Hc, hemocytes; Mg, midgut) were confirmed by quantitative real time PCR. Immunocyte count in hemolymph. **(C)** PO activity of hemolymph (healthy locusts). **(D)** PO activity of hemolymph (infected locusts: 8th day post infection). Values (mean ± SE) with the “^∗^”, “^∗∗^”, and “^∗∗∗^” are significantly different (Independent-Samples *T*-test) at α = 0.05, α = 0.01, and α = 0.001, respectively. HBL, healthy locusts of black phenotype; HLL, healthy locusts of light phenotype; IBL, infected locusts of black phenotype; ILL, infected locusts of light phenotype.

Phenyl Oxidase is required for melanization, which is a form of humoral immunity (**Figure 5A**). We found that the black phenotype had a higher hemolymph PO activity than the light phenotype, both in infected (*t* = 4.961, df = 8, *P* = 1.105 E-3) (**Figure 5D**) and uninfected locusts (*t* = 4.496, df = 8, *P* = 2.012 E-3) (**Figure 5C**). The expression of *serpin* (GenBank: KC118978.1) showed significant differences between the two morphs both in fat bodies (*t* = -3.216, df = 3, *P* = 0.049) and in hemocytes (*t* = -6.047, df = 1.926, *P* = 0.0287) (**Figure 5B**). In the midgut, *serapin* levels were low and there was no significant difference in levels between black and light-colored locusts (**Figure 5B**).

### Healthy Black *L. migratoria* Nymphs Lightened When Exposed to Infected Locusts

**FIGURE 6:**
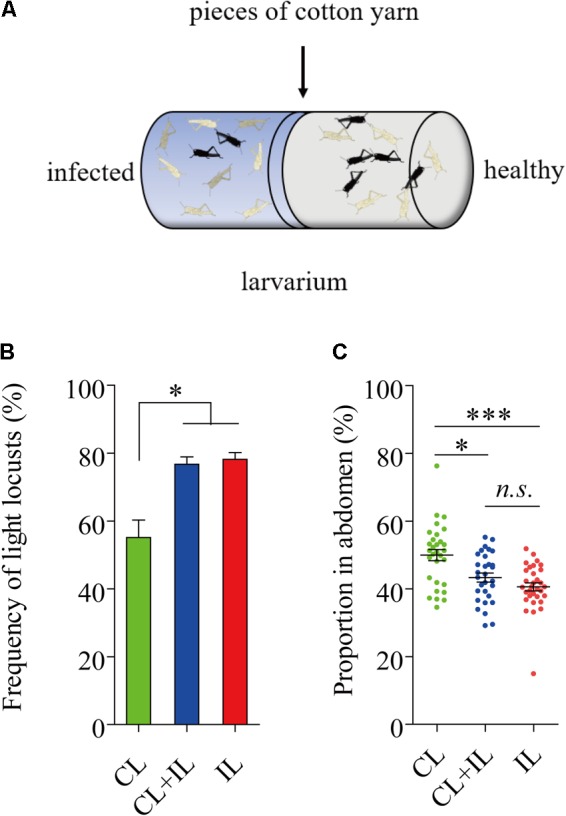
Impact of locusts infected with *P. locustae* on body color of healthy *L. migratoria* nymphs. **(A)** The diagram of polyculture. CL, control locusts (*N* = 30); CL + IL = healthy *L. migratoria* nymphs of laboratorial population reared beside infected locusts (*N* = 30, CL: IL = 1:1); IL = infected locusts (*N* = 32). **(B)** The number of light locusts in the 4th instar. Values (mean ± SE) with the “^∗^” are significantly different (*P* ≤ 0.05) from the untreated controls (infected 0 spores/locust) (Tukey’s Multiple Comparison Test). **(C)** The proportion of the abdomen covered in dark stripes in 5th instar nymphs: the total breadth of dark stripes compared to total abdomen breadth. Values (mean ± SE) with the “^∗∗∗^” are significantly different (*P* ≤ 0.001) (Kruskal–Wallis test). The locusts used in the experiment were normal laboratory population including black light and intermediate phenotypes.

Uninfected locusts were reared with infected locusts for 16 days in cages where locusts were near each other but were kept separate by pieces of cotton yarn (**Figure 6A**). None of the locusts reared on the healthy side were infected by *P. locustae* in the process of experiment. The results showed that the number of black locusts in the uninfected population declined after 8 days (fourth instar) (**Figure 6B**). Additionally, the darkened proportion of the abdomen in fifth-instar nymphs clearly decreased (**Figure 6C**).

### Light Phenotypes Were Conducive to the Propagation and Spread of Microsporidia

**FIGURE 7:**
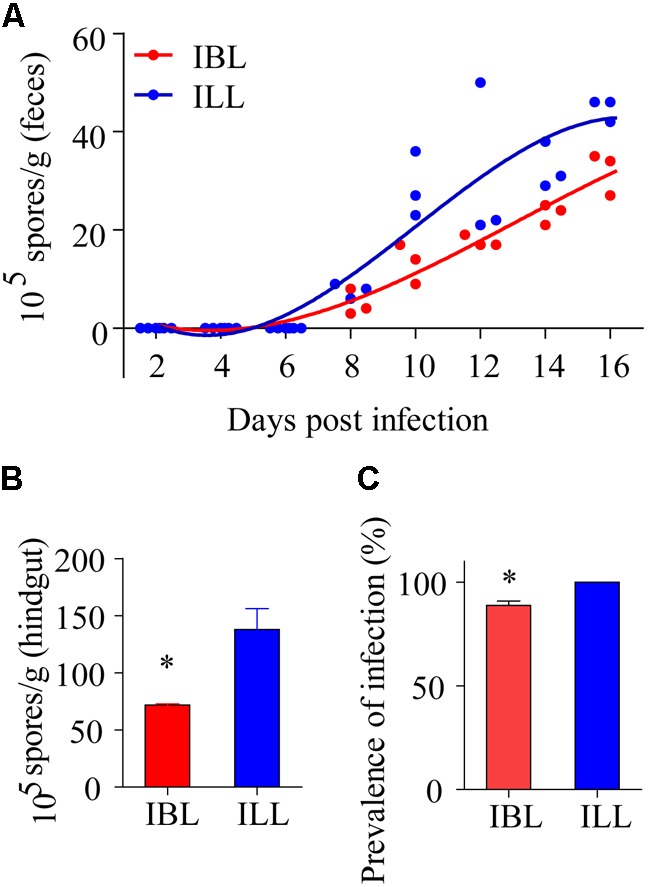
The effects of two phenotypes on propagation and spread of *P. locustae* in the locusts population. **(A)** Number of microsporidia spores contained in the feces of locusts at various days after being infected with 2 × 10^5^ spores of *P. locustae.* **(B)** Number of microsporidia spores contained in the hindguts of locusts infected with *P. locustae.* **(C)** The infection rate of healthy locusts (HL) reared with sick locusts (SL) infected with *P. locustae*. N (HL): N(SL) = 13:13. Values (mean ± SE) with the “^∗^” is significantly different (Independent-Samples *T*-test) at α = 0.05. IBL, infected locusts of black phenotype; ILL, infected locusts of light phenotype.

After locusts were infected with 2 × 10^5^ spores of *P. locustae*, spores were first detected in their feces on the 8th day after infection and the number of spores in the feces then increased with time (**Figure 7A**). The number of microsporidia found in the light phenotype was higher than that in the black phenotype (*t* = 2.824, df = 7, *P* = 0.0256) (two-tailed, paired *T*-test) (**Figure 7A**). In addition, the number of spores retrieved from the hindguts of black locusts was lower than that of light-colored ones on the 16th day postinfection with doses of 2 × 10^5^ *P. locustae* spores/locust (*t* = -3.371, df = 3, *P* = 0.043) (**Figure 7B**). The infection rate of healthy locusts reared with light-colored locusts infected with *P. locustae* was 100% (**Figure 7C**). The infection rate of healthy locusts reared with the infected black phenotype was slightly, but significantly (*t* = -5.283, df = 2, *P* = 0.034) lower than that of healthy locusts reared with the infected light forms at 16 days post-infection (*t* = -5.283, df = 2, *P* = 0.034) (**Figure 7C**).

## Discussion

*Paranosema locustae* is a gut parasite that has been applied widely in the control of grasshoppers in many parts of the world. Usually, *P. locustae* is transmitted horizontally via passive methods under natural conditions but in the current study, an active transmission strategy of *P. locustae* has been demonstrated. First, *P. locustae* infection resulted in a lighter cuticular color in *Locusta migratoria*: normally only a small proportion of gregarious locusts are pale with most either being partly or mostly black (third instar nymphs); but locusts infected with *P. locustae* became pale. And it was found that the change to pale occurred even among uninfected black and partly black nymphs exposed to the infected locusts. When examined for infection, it was found that initially uninfected nymphs had picked up *P. locustae* infections from being exposed to infected nymphs. Clearly, *P. locustae* has the capacity to reduce pigmentation in the locust integument and to pass this reduction in pigmentation on to other healthy individuals in the same population.

However, to counteract the ability of *P. locustae* infections to pass from one individual to another, *L. migratoria* isolate infective individuals in that healthy gregarious locusts in groups march ahead, leaving the solitary pale individuals infected by *P. locustae* behind (Shi et al., 2014). This mechanism of the locust trying to isolate themselves from infected individuals is an adaptation to counter the ability of *P. locustae* to readily pass from one locust to another suggesting that there have been adaptations and counter adaptations by parasite and host in this host parasite relationship.

It is well known that variations in locust body color are related to their density (Maeno and Tanaka, 2008). Some articles have reports of ambient temperature and background colors leading to variations in insect body color (Goulson, 1994; Valverde and Schielzeth, 2015), and some studies have demonstrated that *P. locustae* infection observably affected both behavior and morphological phase transformation (Fu et al., 2010; Feng et al., 2015). But mechanisms by which pathogens change host colors have rarely been reported. We now show that infection by *P. locustae* parasites can alter locust body color.

Changes in skin color in higher animals due to disease are a general phenomenon that indicates immunopathological effects. As in our study, Dubovskiy et al. (2013) reported that the melanic morph was more resistant to gut parasites than the non-melanic morph, and they related the higher resistance to its higher expression of immunity genes, higher number of hemocytes, and up-regulated cuticle Phenyl Oxidase (PO) activity. The more melanin insects have in the cuticle, the healthier they are (Liu et al., 2015). Similarly, human patients with leukoderma are generally plagued by immune disorders, especially autoimmune diseases (Strassner and Harris, 2016).

Several gregarious Lepidopteran species display lower hemocyte counts and decreased PO activity (Wilson et al., 2003). Here, higher PO activity in the black morph led to lower mortality after infection with *P. locustae*. Some articles reported that enhanced activity of prophenoloxidase (PPO), the precursor of PO, and PO can enhance defense against pathogenic microorganisms (Crawford et al., 2012; Binggeli et al., 2014). Usually, inner melanization and exterior pigmentation are closely related, because they result from the same metabolic pathway (Lee et al., 2015). We discovered that light-colored locusts were more sensitive to the microsporidian pathogen than dark-colored individuals, because of their weaker melanization. PO activity in pale locusts was lower, but serpin expression higher than that of black locusts. The increase in pale colored locusts clearly promoted the prevalence of *P. locustae* in the total population. Infection by *P. locustae* led to a decline in PO soon after infection and there was a decline in the expression of the *henna* and *pale* genes: a reduction in these melanization factors might contribute to increase susceptibility in the locust. Overall, these results elucidated a new strategy of positive self-transmission in *P. locustae*.

Mother Nature always grants wisdom to her creatures and feeds them carefully. This wisdom is particularly apparent in the relationships between two interacting species. Here, our team focused on the interacting species *L. migratoria* and *P. locustae*. In a previous study, *L. migratoria* was shown to isolate infective individuals against the spread of *P. locustae* (Shi et al., 2014). The results of this study demonstrate that *P. locustae* promotes pigmentation loss in *L. migratoria*, leading to a greater proportion of light-colored individuals, and these lighter individuals possess weaker immunity against the pathogens. In other words, the increase in light individuals in the locust population weakened their capacity to defend against pathogens or adversity. The number of microsporidia propagated in light-colored locusts after infection was greater than that in black locusts. Thus, the pigmentation loss caused by *P. locustae* promoted an increased prevalence of infection in the overall population. This result is likely to be of ecological and economic significance in enhancing the control of *L. migratoria*.

The horizontal transmission of fungal conidia from infected to healthy individuals has been demonstrated (Dimbi et al., 2013). Nevertheless, numerous transmission routes are considered passive modes, which depend on natural conditions (Peng et al., 2011). In conclusion, we report here a positive transmission strategy and mechanism in *P. locustae* that benefit its propagation and spread. These extraordinary abilities of *P. locustae* have evolved over millennia of years of host-pathogen interaction.

## Author Contributions

ST contributed to constructing the experiment and collecting the data. YW, PL, YG, AL, and YX helped with experiments. WS and ST wrote the manuscript. ST and WS contributed to analyzing the data. WS supervised the work. DH gave some advice on how to do the study and helped to write the manuscript.

## Conflict of Interest Statement

The authors declare that the research was conducted in the absence of any commercial or financial relationships that could be construed as a potential conflict of interest.
