# Supplementary material for: Increase of Albinistic Hosts Caused by Gut Parasites Promotes Self-Transmission
Source: Front Microbiol. 2018 Jul 10;9:1525. doi: 10.3389/fmicb.2018.01525 (PMC6048391; doi:10.3389/fmicb.2018.01525)
Supplement: Supplementary file 1 [file Data_Sheet_1.docx]

**Supplementary Information**

**Increase of albinistic hosts caused by gut parasites promotes self-transmission**

**Shuqian Tan^1^*, Yang Wang^1^, Pingping Liu^1^, Yang Ge^1^, Aomei Li^1^, Yongjie Xing^1^, David M. Hunter^2^ &** **Wangpeng Shi^1^***

1 Department of Entomology and MOA Key Lab of Pest Monitoring and Green Management, College of Plant Protection, China Agricultural University, Beijing, China; 2 125 William Webb Drive, McKellar, Canberra ACT 2617, Australia

Corresponding author: Shu-qian Tan, Wang-peng Shi

Department of Entomology and MOA Key Lab of Pest Monitoring and Green Management, College of Plant Protection, China Agricultural University, Beijing, China

e-mail: [zhiweijianniyixiao@163.com](mailto:zhiweijianniyixiao@163.com), [wpshi@cau.edu.cn](mailto:wpshi@cau.edu.cn)


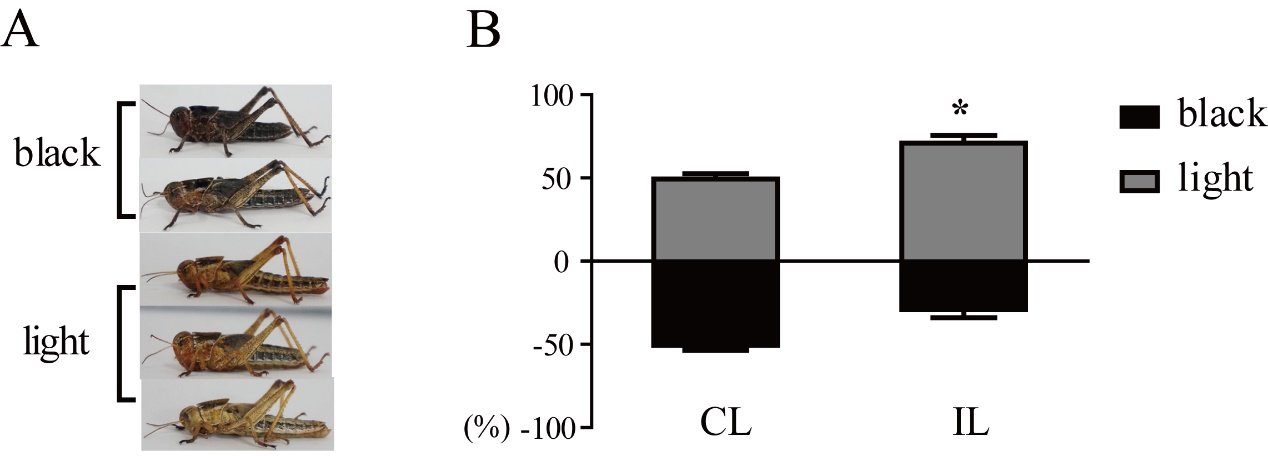


Figure S1 **The variation of locust body color in experimental populations (16th day).** A: Photo of variations in locust body color. black: the locusts identified into shade group; light: the locusts identified into hypochromic group; B: the proportion of black or light locusts in experimental populations (16^th^ day). Values (mean ± SE) with the “*” are significantly different (P ≤ 0.05) (Independent-Samples T test). CL=control locusts; IL=infected locusts.


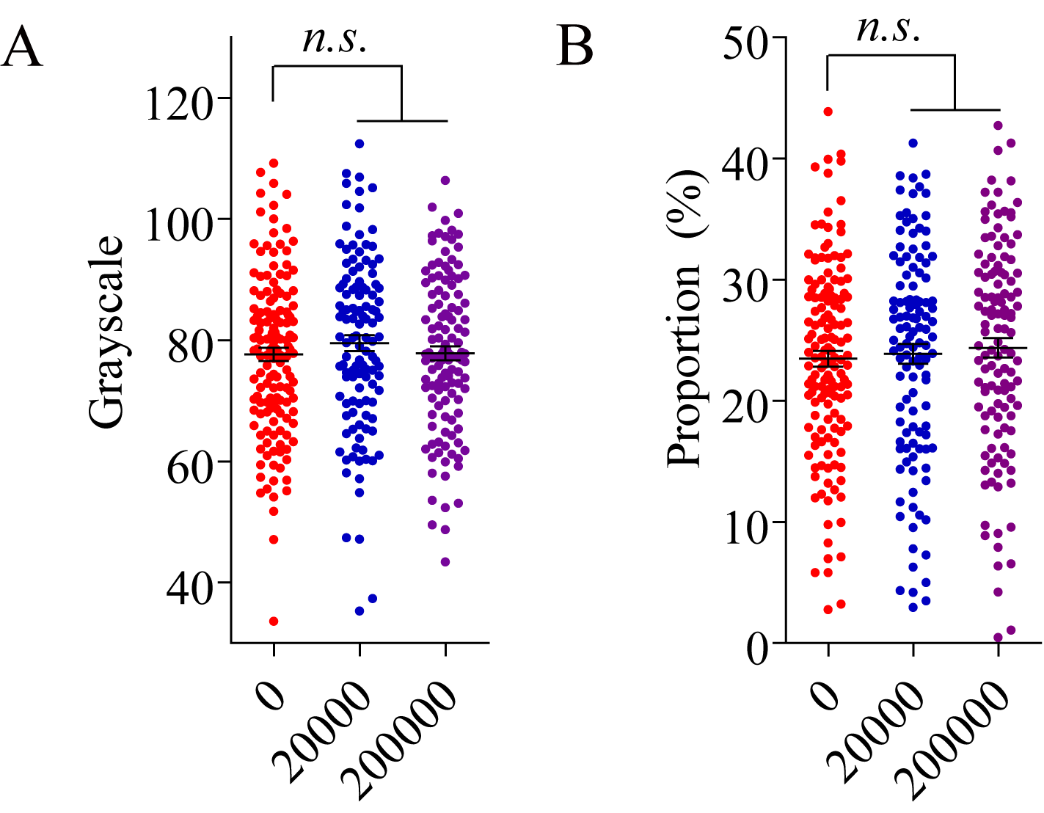


Figure S2 **The variation of dark spots on the locust pronotum (16^th^ day).** A: the [gray](javascript:void(0);) [level](javascript:void(0);) of pronotum (16**^th^** day, fifth instar). B: the proportion of the pronotum covered in spots (16**^th^** day, , fifth instar). Values (mean ± SE) with the “*n.s.*” are not significantly from ck (infected 0 spores/locust) (P ≥ 0.05) (Kruskal-Wallis test)

**Table S1 Impact of *P. locustae* infection on total melanin (TM) in nymphs**

|  |  | total melanin (TM), OD/g | |
| --- | --- | --- | --- |
| Instar | Inoculation dose | thorax | abdomen |
| Fourth instar  (8 day post-inoculation) | Untreated | 8.00±1.41a | 6.50±0.25ab |
|  | 2000 pores/locust | 6.28±1.31a | 7.61±0.69a |
|  | 20000 pores/locust | 6.58±0.72a | 5.17±0.33c |
| Fifth instar  (16 day post-inoculation) | Untreated | 33.25±5.40a | 32.66±7.17a |
|  | 2000 pores/locust | 19.50±1.87a | 24.33±1.62a |
|  | 20000 pores/locust | 19.08±2.61a | 20.00±0.67a |

Data are mean ± SE levels of total melanin (TM) in integument of fourth and fifth instar nymphs after inoculation with *P. locustae*. Any two treatments with the same lower-case letter are not significantly different at α = 0.05 (one-way ANOVA).

**Table S2 Impact of *P. locustae* infection on alkali solubility of melanin (ASM) in nymphs**

|  |  | alkali solubility of melanin (ASM), OD/g | |
| --- | --- | --- | --- |
| Instar | Inoculation dose | thorax | abdomen |
| Fourth instar  (8 day post-inoculation) | Untreated | 29.21±5.44a | 84.13±17.17a |
|  | 2000 pores/locust | 18.33±0.60a | 47.21±9.52ab |
|  | 20000 pores/locust | 22.21±5.24a | 20.21±5.33b |
| Fifth instar  (16 day post-inoculation) | Untreated | 26.58±0.62a | 51.17±7.97a |
|  | 2000 pores/locust | 21.79±0.73b | 39.50±1.99a |
|  | 20000 pores/locust | 21.50±2.17b | 31.46±2.98a |

Data are mean ± SE levels of alkali solubility of melanin (ASM) in integument of fourth and fifth instar nymphs after inoculation with *P. locustae*. Any two treatments with the same lower-case letter are not significantly different at α = 0.05 (one-way ANOVA).

**Table S3. Primers for Quantitative RT-PCR validation of the genes in dopamine pathway and immunogens (Ma et al., 2011;** **Wang et al., 2013)**

| Genes | Sequence （5’🡪3’） |
| --- | --- |
| *β-actin* | CGAAACCTTTAATACCCCAG |
|  | CCATCACCAGAATCCAACAC |
| *Pale* | AATCCTGCTTACGTGCTGCT |
|  | TGTAGCATTTAGGCGGAGGT |
| *Henna* | CGGTTCCCTAGCCAGTATGA |
|  | CGTGGAAACCAAGGAACAGT |
| *Serpin* | TGAATCGCATAGGTTTGAAGA |
|  | TCGCCTCTGAAGTAAATAGCA |

**References**

Ma Z, [Guo W](https://www.ncbi.nlm.nih.gov/pubmed/?term=Guo%20W%5BAuthor%5D&cauthor=true&cauthor_uid=21325054), [Guo X](https://www.ncbi.nlm.nih.gov/pubmed/?term=Guo%20X%5BAuthor%5D&cauthor=true&cauthor_uid=21325054), [Wang X](https://www.ncbi.nlm.nih.gov/pubmed/?term=Wang%20X%5BAuthor%5D&cauthor=true&cauthor_uid=21325054), [Kang L](https://www.ncbi.nlm.nih.gov/pubmed/?term=Kang%20L%5BAuthor%5D&cauthor=true&cauthor_uid=21325054). 2011. Modulation of behavioral phase changes of the migratory locust by the catecholamine metabolic pathway. Proceedings of the National Academy of Sciences 108: 3882-3887. http://doi.org/10.1073/pnas.1015098108.

Wang Y, Yang P, Cui F, Kang L. 2013. Altered Immunity in Crowded Locust Reduced Fungal (*Metarhizium anisopliae*) Pathogenesis. PLoS Pathogens 9 (1): e1003102. http://doi.org/10.1371/journal.ppat.1003102
